# Design and Investigation of the High Performance Doping-Less TFET with Ge/Si_0.6_Ge_0.4_/Si Heterojunction

**DOI:** 10.3390/mi10060424

**Published:** 2019-06-24

**Authors:** Tao Han, Hongxia Liu, Shupeng Chen, Shulong Wang, Wei Li

**Affiliations:** Key Laboratory for Wide-Bandgap Semiconductor Materials and Devices of Education, The School of Microelectronics, Xidian University, Xi’an 710071, China; taohan373@gmail.com (T.H.); slwang@xidian.edu.cn (S.W.); li20101467@163.com (W.L.)

**Keywords:** DLTFET, on-state current, subthreshold swing, frequency characteristics

## Abstract

A high performance doping-less tunneling field effect transistor with Ge/Si_0.6_Ge_0.4_/Si heterojunction (H-DLTFET) is proposed in this paper. Compared to the conventional doping-less tunneling field effect transistor (DLTFET), the source and channel regions of H-DLTFET respectively use the germanium and Si_0.6_Ge_0.4_ materials to get the steeper energy band, which can also increase the electric field of source/channel tunneling junction. Meanwhile, the double-gate process is used to improve the gate-to-channel control. In addition, the effects of Ge content, electrode work functions, and device structure parameters on the performance of H-DLTFET are researched in detail, and then the above optimal device structure parameters can be obtained. Compared to the DLTFET, the simulation results show that the maximum on-state current, trans-conductance, and output current of H-DLTFET are all increased by one order of magnitude, whereas the off-state current is reduced by two orders of magnitude, so the switching ratio increase by three orders of magnitude. At the same time, the cut-off frequency and gain bandwidth product of H-DLTFET increase from 1.75 GHz and 0.23 GHz to 23.6 GHz and 4.69 GHz, respectively. Therefore, the H-DLTFET is more suitable for the ultra-low power integrated circuits.

## 1. Introduction

The performance of integrated circuits (ICs) has been greatly improved with the size of the metal oxide semiconductor field effect transistors (MOSFETs) continues to decrease, while the MOSFETs are more susceptible to the short channel effects and quantum effects [1,2,3]. The MOSFETs based on the physical mechanism of thermal electron excitation have the subthreshold swing (SS) limit of 60 mV/Dec, the smaller switching ratio, the higher off-state current and the larger power consumption at room temperature, which cannot meet the requirements of the ultra-low power ICs [4,5]. The performance and power consumption of the MOSFETs are severely degraded when the threshold voltage of the device is reduced [6]. Therefore, it is necessary to explore the new low-power electronic devices with the smaller SS and higher switching ratio.

In recent years, many scholars have conducted extensive research on various new materials, new structures, and new physical mechanisms of device work [7,8,9]. Different from the MOSFETs, the working mechanism of tunneling field effect transistors (TFETs) is based on the quantum effect of electron Band-To-Band Tunneling (BTBT). The TFETs have the lower off-state leakage current, higher robustness of the short channel effect and faster switching speed, which is compatible with CMOS processes [10,11]. The TFET can break the SS limit of 60 mV/Dec, which can obtain the better device performance and lower static power consumption under the lower voltage operating conditions [12]. At the same time, it can also effectively improve circuit performance and reduce circuit power consumption, which can make it a potential device in the ultra-low power ICs [13]. However, the on-state current of the conventional silicon based TFET is smaller. The reason is that the carrier tunneling quality and the forbidden band width are so larger that the electrons tunneling probability is smaller. Meanwhile, the SS of TFET cannot also reach the ideal value due to the non-ideal effects, such as the complex process of ultra-steep PN junction. To improve the on-state current and SS of TFETs, it is necessary to further increase the gate-to-channel control by the new materials and processes [14,15,16].

A high performance doping-less tunneling field effect transistor with Ge/Si_0.6_Ge_0.4_/Si heterojunction (H-DLTFET) is proposed in this paper. Compared to the conventional DLTFET, the source and channel regions of the H-DLTFET respectively use the germanium and Si_0.6_Ge_0.4_ materials. To make the energy band becomes steeper, source region use the narrow bandgap semiconductor material germanium, which can also effectively improve the electric field of source/channel tunneling junction. The electrons from the valence band of source region are easier to tunnel into the conduction band of channel region, which can increase the on-state current effectively [17]. The channel region uses Si_0.6_Ge_0.4_ material to improve the channel carrier mobility. Meanwhile, the SS and frequency characteristics of H-DLTFET can be greatly improved. Besides, the dual gate process is used to improve the gate-to-channel control, which can effectively reduce the adverse effects of short channel effects [18]. Meanwhile, the stronger gate control can provide the higher saturation current density and lower off-state leakage current [19]. The main research contents of this paper are as follows: First, the simulation structure and models of DLTFET and H-DLTFET are introduced. Next, the input and output characteristics of DLTFET and H-DLTFET are analyzed. Then, the working mechanism of H-DLTFET is described. Afterwards, effects of Ge content, electrode work function, and structure parameters on the performance of H-DLTFET are researched systematically. Subsequently, the C-V and frequency characteristics of DLTFET and H-DLTFET are compared. Finally, the simulation results show that the H-DLTFET has the greater potential in the low-power ICs.

## 2. Device Structure and Models

To improve the on-state current and SS of conventional DLTFET, the differences of H-DLTFET are that the germanium and Si_0.6_Ge_0.4_ materials are used in the source and channel regions, respectively. Then, the gate-to-channel control capability can be improved by enhancing the coupling between gate and back-gate. Finally, the H-DLTFET can obtain the higher on-state current by adjusting the length and height of effective channel tunneling region.

**Figure 1:**
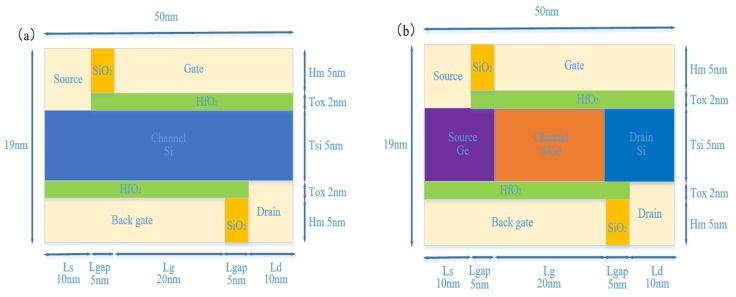
The schematic of (**a**) conventional DLTFET, (**b**) new proposed H-DLTFET.

Figure 1a,b respectively show the schematic of the conventional DLTFET and the new proposed H-DLTFET. The specific parameters are as follows: channel thickness *H_c_* = 5 nm; gate oxide thickness *T_ox_* = 2 nm; the length of source region (*L_s_*), gap region (*L_gap_*), channel region (*L_g_*), and drain region (*L_d_*) are 10 nm, 5 nm, 20 nm, and 10 nm, respectively. Meanwhile, the doping concentration and type of *L_s_*, *L_gap_*, *L_g_*, and *L_d_* are 1 × 10^18^ cm^−3^ and n-type, respectively. Besides, the work functions of gate, source, drain, and back-gate electrodes are respectively 4 eV, 5.3 eV, 4.2 eV, and 5.1 eV.

All simulation results in this paper are based on the SILVACO TCAD simulation software (Santa Clara University, Santa Clara, CA, USA). It is known that the spatial distribution variation of band structure and the narrowing effect of forbidden band width have an effect on the non-local Band-To-Band tunneling probability of carriers [20]. Due to the existence of traps, the Shockley-Read-Hall composite and the non-local Band-To-Band tunneling models are used in this paper. The Lombardi mobility model is also considered to accurately calculate the effects of acoustic phonon scattering and surface roughness on the mobility of carriers [21]. In addition, there are also included the Fermi-Dirac statistics and the Band-Gap Narrowing model, which can be explained by the presence of heavily doped regions.

## 3. Discussion of Simulation Results

### 3.1. The Input Characteristics

**Figure 2:**
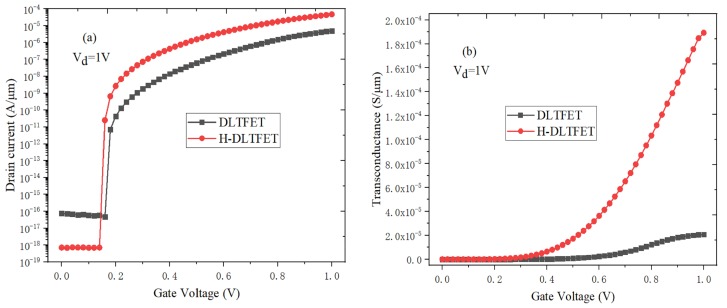
(**a**) Transfer characteristic, (**b**) Trans-conductance of DLTFET and H-DLTFET.

Figure 2a shows the transfer characteristics of DLTFET and H-DLTFET. The minimum off-state leakage currents of DLTFET and H-DLTFET are respectively 7.46 × 10^−17^ A/μm and 7.14 × 10^−19^ A/μm, whereas the maximum on-state currents of DLTFET and H-DLTFET are 5.03 × 10^−6^ A/μm and 4.71 × 10^−5^ A/μm, respectively. The off-state current of H-DLTFET decreases by two orders of magnitude compared with the conventional DLTFET. And the on-state current and switching ratio are respectively increased by an order of magnitude and three orders of magnitude at the same operating voltage. The reason is that a large number of electron-hole pairs from source region can tunnel into the channel, and the tunneling current would increase exponentially with the electric field. However, the electrons of channel center are depleted and the on-state current is saturated when gate voltage continues to increase. Meanwhile, the average SS and point SS of H-DLTFET respectively decrease from 41.5 mV/Dec and 4 mV/Dec to 15.7 mV/Dec and 2.6 mV/Dec. It can be concluded that the H-DLTFET have the larger on-state current, higher switching ratio and smaller SS than that of DLTFET, so it has the greater potential in the ultra-low power ICs.

The trans-conductance (*g_m_*) is an important characterization parameter of frequency characteristics, which can determine the intrinsic gain of semiconductor devices. Figure 2b shows the *g_m_* value of DLTFET and H-DLTFET as a function of gate voltage. The *g_m_* can be calculated by the following Equation (1) [22]:

(1) $$g_{m}=dI_{DS}/dV_{GS}$$

The *g_m_* value mainly depends on the output leakage current, and the faster the increase rate of output leakage current is, the larger the *g_m_* is. The *g_m_* values of DLTFET and H-DLTFET increase with the gate voltage increases. This is because the barrier width of tunneling junction decreases, the tunneling electrons increase, and the output leakage current increases gradually. It can be seen from Figure 2b that the *g_m_* values of DLTFET and H-DLTFET are respectively 2.07 × 10^−5^ S/μm and 1.89 × 10^−4^ S/μm. Due to the higher on-state current, the maximum *g_m_* value of H-DLTFET can be increased by an order of magnitude.

### 3.2. The Output Characteristics

**Figure 3:**
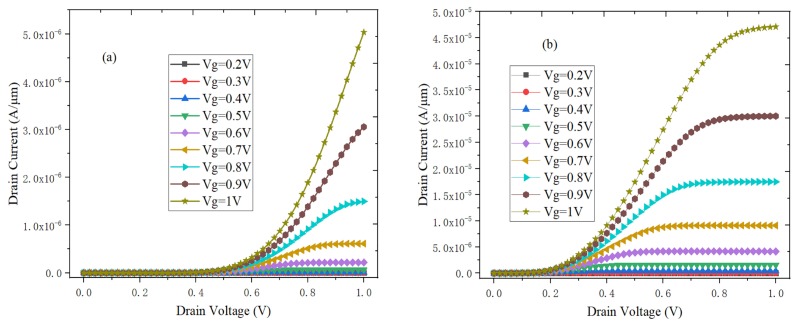
The output characteristics of (**a**) DLTFET, (**b**) H-DLTFET.

Figure 3a,b respectively show the output characteristics of DLTFET and H-DLTFET under the different gate voltages. The output currents of DLTFET and H-DLTFET can be divided into the exponential growth region and the saturation region. The output current would increase to the saturation value when the drain-source voltage is greater than the saturation pinch-off voltage. At the same time, there is no change in the tunneling barrier width. Besides, the output current would increase when the gate voltage increase. The reason is that the energy band of tunneling junction could be steeper, and the BTBT distance decrease with the gate voltage increases. The maximum output currents of DLTFET and H-DLTFET are respectively 5.03 × 10^−6^ A/μm and 4.71 × 10^−5^ A/μm. The output current of H-DLTFET can be increased by an order of magnitude compared with the DLTFET, so the H-DLTFET has the higher saturation output current.

### 3.3. The Operating Mechanism of H-DLTFET

**Figure 4:**
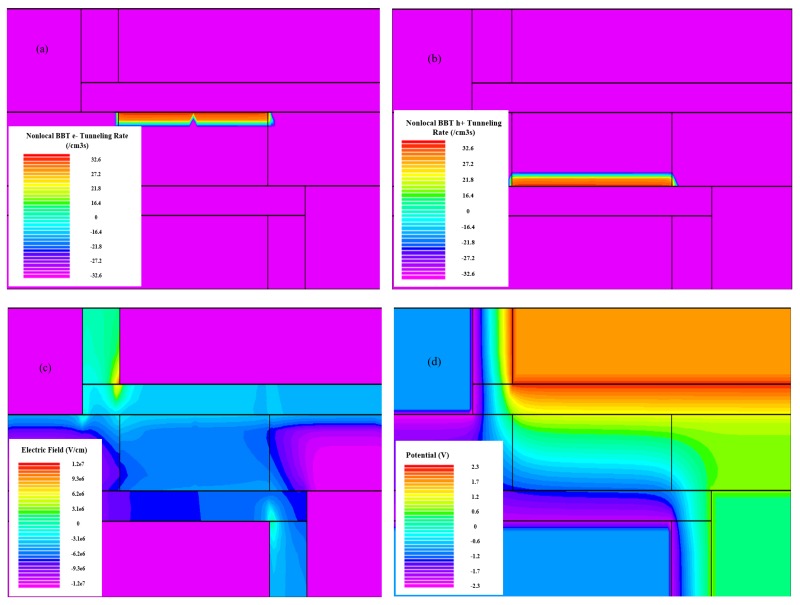
(**a**) e-BTBT rate; (**b**) h-BTBT rate; (**c**) Electric field; and (**d**) Potential distribution of the H-DLTFET.

**Figure 5:**
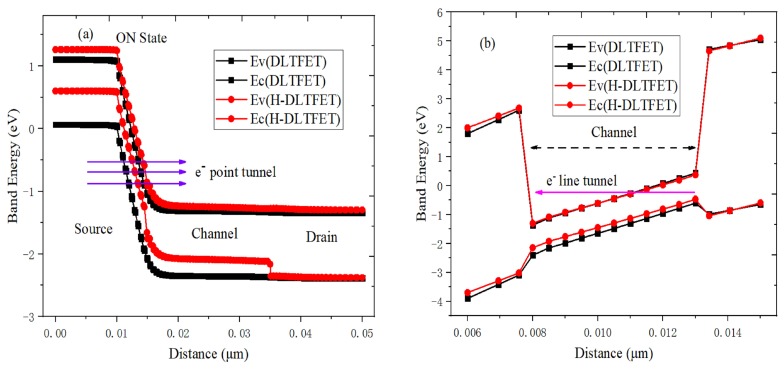
On-state energy band of DLTFET and H-DLTFET (**a**) from source to drain; (**b**) from channel top to channel bottom.

Figure 4 and Figure 5 respectively illustrate the operating mechanism of H-DLTFET. Figure 4a,b respectively show the electron BTBT and hole BTBT efficiencies of the H-DLTFET channel under the on-state condition. It can be found that the e-BTBT region occurs near the channel top, and the h-BTBT region is mainly concentrated on the channel bottom. The line tunneling current is formed in the overlapping region between gate and back gate, where the electric field is parallel to the e- and h- BTBT tunneling directions [23]. In addition, the point tunneling also exists in H-DLTFET, which can be explained by the higher BTBT efficiency of the source-channel tunneling junction. The reason the tunneling current increase is that the area of the line tunneling is larger than that of the point tunneling. The electric field and potential distributions of H-DLTFET are respectively shown in Figure 4c,d. Due to the higher electric field and potential of the source-channel tunneling junction, electrons from the electron doped source region and the channel bottom region are more easily absorbed by the drain region, which would result in the higher on-state current [24].

Figure 5a,b respectively show the on-state energy band of DLTFET and H-DLTFET under the different position. It can be observed from Figure 5a that the energy band at the source-channel tunneling junction of H-DLTFET is more curved than that of DLTFET, so it has the narrower tunneling barrier width. The electrons from source region can tunnel to the channel region easily, which is largely dependent on the tunneling barrier width [25]. Besides, the line tunneling current of DLTFET and H-DLTFET can also be confirmed by the on-state energy band from top channel to bottom channel, as shown in Figure 5b. Since the valence band of the channel bottom can be aligned with the conduction band of the channel top, many electrons from the channel bottom can tunnel to the channel top, thereby forming the tunneling current.

### 3.4. Effect of the Device Parameters on the Performance of H-DLTFET

**Figure 6:**
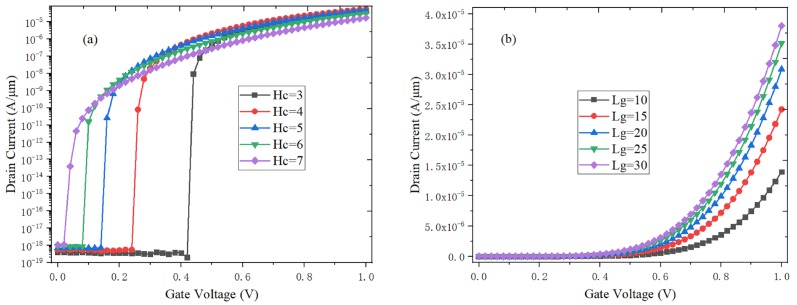
Effect of device parameters on the transfer characteristics of H-DLTFET (**a**) channel thickness *Hc*, (**b**) channel length *Lg*.

In Figure 6a, the channel electric field coupling effect between gate and back gate is weakened when the channel thickness (*Hc*) increase. Meanwhile, the electric field, electronic tunneling probability and on-state current of channel are all reduced. Figure 6b shows the effect of channel length (*Lg*) on the transfer characteristics. The barrier width of the tunneling junction becomes less susceptible to the drain voltage with the *Lg* increases, the line tunneling area and the electron tunneling number increase, thereby increasing the on-state current of H-DLTFET. However, the on-state current rises slowly and the preparation process becomes complicated when the channel length exceeds 20 nm [26,27]. It can be concluded that the optimal *Hc* and *Lg* are 5 nm and 20 nm, respectively.

**Figure 7:**
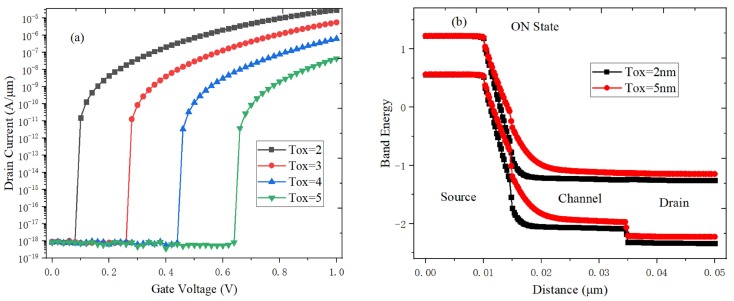
Effect of gate oxide thickness on (**a**) transfer characteristics, (**b**) on-state energy band.

In Figure 7a, both the gate voltage and electric field required for the point tunneling would increase when the gate dielectric thickness (*T_ox_*) increases. At the same time, the electric field at the interface between the gate oxide dielectric and channel is weakened. The on-state current increases from 4.4 × 10^−8^ A/μm to 3.1 × 10^−5^ A/μm, and the switching ratio is almost increased by three orders of magnitude when *T_ox_* decrease from 5 nm to 2 nm. Figure 7b shows the energy band of the source-channel tunneling junction at the different *T_ox_*. As the *T_ox_* decreases, the energy band becomes steeper. Both the electron BTBT probability and the tunneling area are increased, and the gate-to-channel control capability increases, so the on-state current can be effectively improved. In a word, the optimal *T_ox_* is 2 nm.

**Figure 8:**
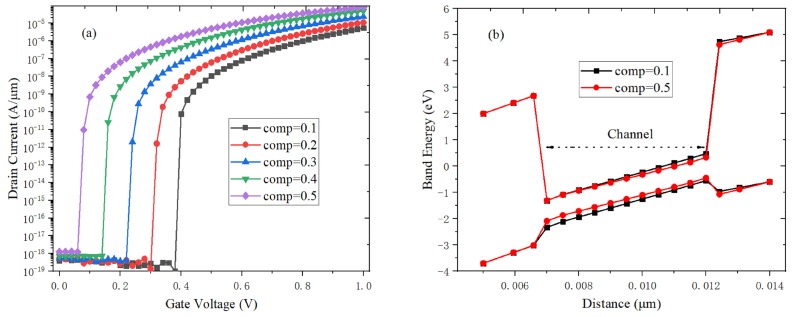
Effect of germanium content on (**a**) transfer characteristics; (**b**) on-state energy band.

It can be found by observing Figure 8a that the on-state tunneling current increases with the germanium content of Si_1-x_Ge_x_ material increases. This is because the germanium material can provide the smaller electron tunneling quality [28]. The number of tunneling electrons increase, and the band gap becomes narrower when the germanium content increases, which can improve the e-BTBT probability and SS characteristics of H-DLTFET. As shown in Figure 8b, the energy band width becomes narrower, and the energy band becomes steeper with the germanium content increases, which result in the higher e-BTBT probability. The H-DLTFET can obtain the relatively smaller off-state current and the larger on-state current when the optimal Ge content is 0.4. Therefore, the channel of H-DLTFET uses the Si_0.6_Ge_0.4_ material.

**Figure 9:**
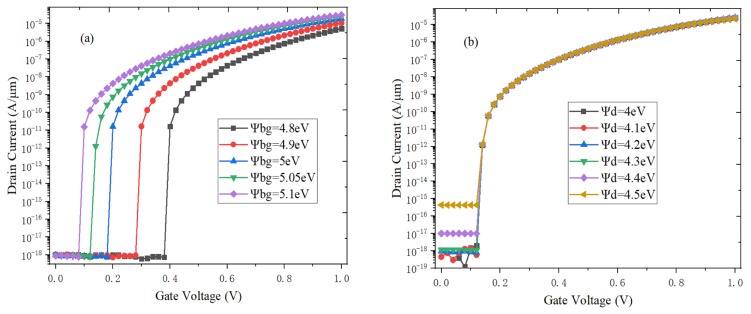

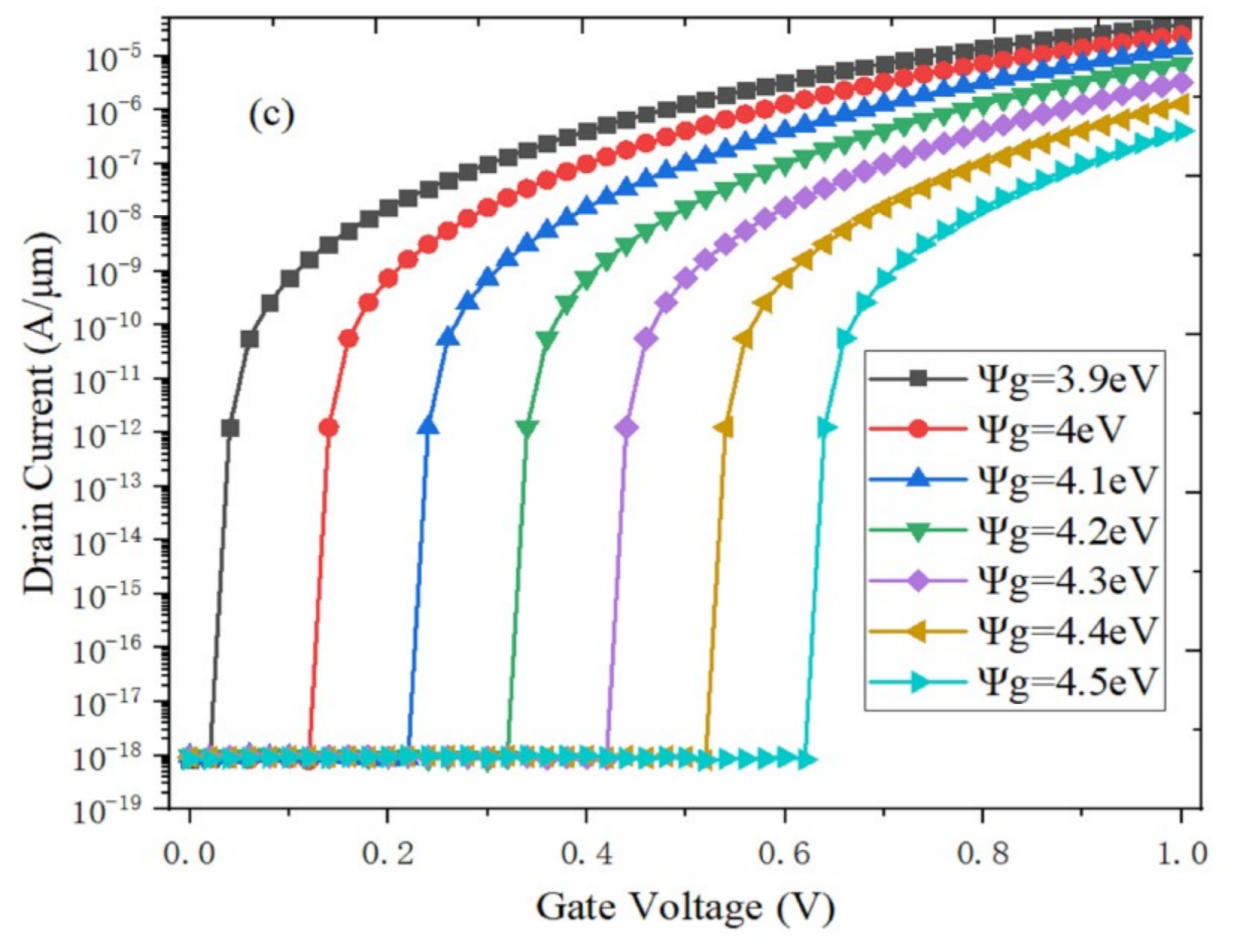
Effect of work function (**a**) back gate *Ψbg*; (**b**) drain *Ψd*; (**c**) gate *Ψg* on the transfer characteristics of H-DLTFET.

In Figure 9a, the on-state current increases and the transfer characteristic curve shifts to the left when the back-gate work function increases. The reason is that a large amount of holes are accumulated in the channel bottom when the back-gate has the higher work function. It can be seen from Figure 9b that the channel barrier height decreases with the drain work function increases, which would result in the higher carrier number and off-state current. The optimal drain work function is 4.2 eV. As shown in Figure 9c, the coupling capability between double gate and channel becomes stronger when the gate work function decreases, so the electric field of drain region increases. The electrons from the valence band of channel region can easily pass through the narrow barrier to the drain region to form the tunneling current [29].

### 3.5. The C-V Characteristics

**Figure 10:**
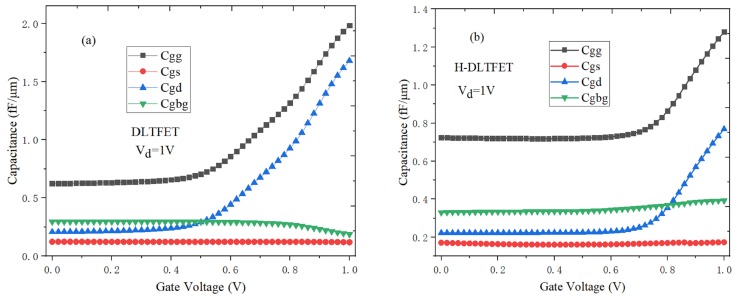
The C-V characteristic of (**a**) DLTFET; (**b**) H-DLTFET.

The gate capacitance (*C_gg_*) is an important indicator for evaluating the frequency characteristics [30]. Figure 10a,b respectively show the C-V characteristics of DLTFET and H-DLTFET at the operating frequency f = 1 MHz. The *C_gg_* mainly includes the gate-drain capacitance (*C_gd_*), gate-source capacitance (*C_gs_*) and gate-back gate capacitance (*C_gbg_*) [31]. It can be seen from Figure 10 that the *C_gd_* increases exponentially with the gate voltage increases. The reason is that the tunneling barrier width decreases when the gate voltage increases, and the inversion layer extending from drain region to source region can be formed. The *C_gs_* is mainly composed of parasitic capacitance, and the order of magnitude is small relative to the *C_gd_* under the inversion layer, which is weakly affected by the bias voltage. Therefore, the *C_gg_* is mainly determined by the *C_gd_* at the high gate voltage. Almost all electrons of H-DLTFET can be collected by the drain region immediately, and the channel electron concentration is lower, which would result in the smaller *C_gg_* and *C_gd_*. It can be seen from Figure 10 that the *C_gg_* and *C_gd_* of H-DLTFET respectively decrease from 1.98 fF/μm and 1.68 fF/μm to 1.28 fF/μm and 0.77 fF/μm compared with the conventional DLTFET, so the H-DLTFET has the better frequency characteristics.

### 3.6. The Frequency Characteristics

As an important indicator, the cut-off frequency is used to evaluate the frequency characteristics of electronic devices. It can be obtained by the ratio of gm to *C_gg_*, and the specific calculation formula is shown in Equation (2) [32].

(2) $$f_{T}=\frac{g_{m}}{2\pi C_{gs}\sqrt{1+2C_{gd}/C_{gs}}}\approx \frac{g_{m}}{2\pi \left(C_{gs}+C_{gd}\right)}=\frac{g_{m}}{2\pi C_{gg}}$$

**Figure 11:**
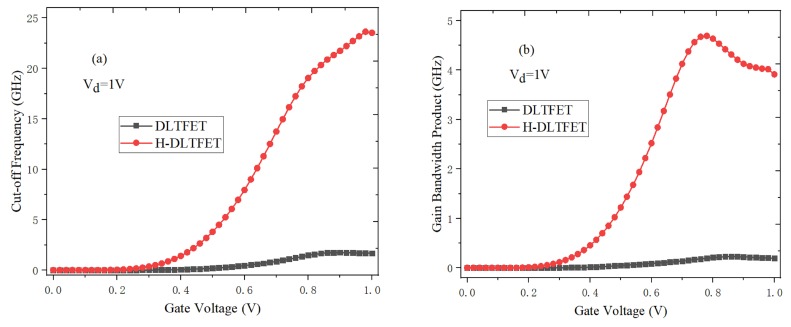
(**a**) Cut-off frequency, (**b**) Gain bandwidth product of DLTFET and H-DLTFET.

In Figure 11a, the cut-off frequency increases when the gate voltage increases. This is because the on-state current and *g_m_* value increase with the electronic BTBT efficiency increases. However, the cut-off frequency has no change or even decreases when the gate voltage continues to increase to the high gate voltage. This is due to the increase of *C_gg_* and the decrease of *g_m_*, which is caused by the mobility degradation. In addition, the cut-off frequency of H-DLTFET is much larger than that of DLTFET, which can be explained by the smaller *C_gg_* of H-DLTFET. The larger the *g_m_* value is, the higher the cut-off frequency is.

The gain bandwidth product is another important indicator in the analysis of frequency characteristics, which can be calculated by the Equation (3) [33,34].

(3) $$GWB=g_{m}/2\pi 10C_{gd}$$

It can be seen from Figure 11b that the gain bandwidth product of H-DLTFET is significantly higher than that of DLTFET. Due to the increase of *g_m_*, the gain bandwidth product initially increases as the gate voltage increases. However, the gain bandwidth product decreases when the gate voltage is greater than 0.8 V. This is because the common impact between the mobility degradation and the parasitic capacitance. In addition, the overall trend of gain bandwidth product as a function of gate voltage is consistent with the cut-off frequency. Compared to the conventional DLTFET, the cut-off frequency and gain bandwidth product of H-DLTFET respectively increase from 1.75 GHz and 0.23 GHz to 23.6 GHz and 4.69 GHz. Therefore, the H-DLTFET has the better frequency characteristics.

## 4. Conclusions

A high performance H-DLTFET is constructed and studied in this paper. Compared to the traditional DLTFET, the differences of H-DLTFET are that the source and channel regions respectively use the narrow band gap semiconductor germanium material and the high carrier mobility Si_0.6_Ge_0.4_ material, the electric field of source/channel tunneling junction increases and the energy band becomes steeper. At the same time, the dual gate process can improve the gate-to-channel control capability. In addition, the effects of germanium content, electrode work function, and device structure parameters on the performance of H-DLTFET are analyzed systematically, and then the above optimal parameters can be obtained to optimize the overall performance of H-DLTFET. Compared to the conventional DLTFET, the simulation results show that the on-state current and switching ratio of H-DLTFET can respectively increase by one order of magnitude and three orders of magnitude, and the off-state current is reduced by two orders of magnitude at the same operating voltage. At the same time, both the maximum *g_m_* and output current are increased by an order of magnitude. The average SS and point SS of H-DLTFET respectively decrease from 41.5 mV/Dec and 4 mV/Dec to 15.7 mV/Dec and 2.6 mV/Dec. And the *C_gg_* and *C_gd_* of H-DLTFET are also decreased from 1.98 fF/μm and 1.68 fF/μm to 1.28 fF/μm and 0.77 fF/μm, respectively. Meanwhile, the cut-off frequency and gain bandwidth product of H-DLTFET respectively increase from 1.75 GHz and 0.23 GHz to 23.6 GHz and 4.69 GHz. Therefore, the H-DLTFET is more suitable for the ultra-low power integrated circuits.
